# Development and Validation of an Automated Algorithm to Detect Atrial Fibrillation Within Stored Intensive Care Unit Continuous Electrocardiographic Data: Observational Study

**DOI:** 10.2196/18840

**Published:** 2021-02-15

**Authors:** Allan J Walkey, Syed K Bashar, Md Billal Hossain, Eric Ding, Daniella Albuquerque, Michael Winter, Ki H Chon, David D McManus

**Affiliations:** 1 Boston University School of Medicine The Pulmonary Center Boston, MA United States; 2 Department of Biomedical Engineering University of Connecticut Storrs, CT United States; 3 University of Massachusetts Medical School Worcester, MA United States; 4 Boston University School of Public Health Boston, MA United States

**Keywords:** atrial fibrillation, sepsis, intensive care unit, big data, data science

## Abstract

**Background:**

Atrial fibrillation (AF) is the most common arrhythmia during critical illness, representing a sepsis-defining cardiac dysfunction associated with adverse outcomes. Large burdens of premature beats and noisy signal during sepsis may pose unique challenges to automated AF detection.

**Objective:**

The objective of this study is to develop and validate an automated algorithm to accurately identify AF within electronic health care data among critically ill patients with sepsis.

**Methods:**

This is a retrospective cohort study of patients hospitalized with sepsis identified from Medical Information Mart for Intensive Care (MIMIC III) electronic health data with linked electrocardiographic (ECG) telemetry waveforms. Within 3 separate cohorts of 50 patients, we iteratively developed and validated an automated algorithm that identifies ECG signals, removes noise, and identifies irregular rhythm and premature beats in order to identify AF. We compared the automated algorithm to current methods of AF identification in large databases, including ICD-9 (International Classification of Diseases, 9th edition) codes and hourly nurse annotation of heart rhythm. Methods of AF identification were tested against gold-standard manual ECG review.

**Results:**

AF detection algorithms that did not differentiate AF from premature atrial and ventricular beats performed modestly, with 76% (95% CI 61%-87%) accuracy. Performance improved (*P*=.02) with the addition of premature beat detection (validation set accuracy: 94% [95% CI 83%-99%]). Median time between automated and manual detection of AF onset was 30 minutes (25th-75th percentile 0-208 minutes). The accuracy of ICD-9 codes (68%; *P*=.002 vs automated algorithm) and nurse charting (80%; *P*=.02 vs algorithm) was lower than that of the automated algorithm.

**Conclusions:**

An automated algorithm using telemetry ECG data can feasibly and accurately detect AF among critically ill patients with sepsis, and represents an improvement in AF detection within large databases.

## Introduction

Atrial fibrillation (AF) is the most common arrhythmia during critical illness [1]. Among the most common causes of critical illness is sepsis—the potentially life-threatening syndrome caused by a dysregulated response to infection [2]. New-onset AF during sepsis is of special concern, as it is associated with increased mortality [3,4] and stroke risk [5], and likely represents a sepsis-defining organ dysfunction [6]. Despite the associated high morbidity and mortality, few studies have investigated potential mechanisms or optimal treatments of new-onset AF during sepsis. Given that large-scale manual review of continuous electrocardiographic (ECG) recordings is not feasible, and administrative data do not allow identification of AF timing, there has been increasing interest in developing and refining automated algorithms for the detection of AF in electronic health record data that facilitate AF research [7]. However, automated AF detection among critically ill patients with sepsis faces additional challenges, including telemetry data that may be subject to high burdens of premature beats, other arrhythmias, noise [8], and signal loss. Reliable, real-time, automated approaches to accurately identify ECG noise and artifacts are critical to accurate identification of AF in an intensive care unit (ICU) setting and are underdeveloped. We sought to (1) develop, validate, and iteratively evaluate the performance of a novel algorithm that incorporates the critical elements necessary for AF identification during critical illness including noise elimination, premature atrial and ventricular beat detection [9], and AF detection, using a large-scale, electronic health database with standard telemetry ECG data, and (2) compare performance characteristics of automated AF identification with other methods of AF ascertainment within electronic health record data.

## Methods

### Cohort

We identified adult patients with sepsis defined by ICD-9 (International Classification of Diseases, 9th edition) codes for infection and acute organ dysfunction as described previously [10] using Medical Information Mart for Intensive Care (MIMIC III) open source medical record data [11]. MIMIC III is a single-center database from a large tertiary care hospital, with linked ECG telemetry waveform and electronic medical record information from patients hospitalized between 2001 and 2012. Patients without a linked waveform file, with a paced rhythm, with absent or corrupted ECG recordings, with fewer than 6 hours of ECG telemetry data, or with more than 55 hours of ECG telemetry data were excluded from the analysis.

### Waveform Selection and Gold-Standard Rhythm Status Determination

We performed iterative training and testing of automated AF detection algorithms. We selected 25 candidate case patients with AF during sepsis and 25 candidate control patients without AF during sepsis as identified by ICD-9 codes (427.31). The 50 candidate waveforms were then reviewed manually by trained study staff (DA and ED) with the final adjudication of rhythm status (sinus rhythm vs AF) by a board-certified clinical cardiac electrophysiologist (DM) as the gold standard [12]. The 50 candidate waveforms were sent to the algorithm development team for adjudication of rhythm status via the automated algorithm. Investigators involved with algorithm development and testing (MH, SB, and KC) were blinded to each patient’s gold-standard rhythm determination (sinus rhythm or AF).

### Automated AF Detection Algorithm

**Figure 1:**
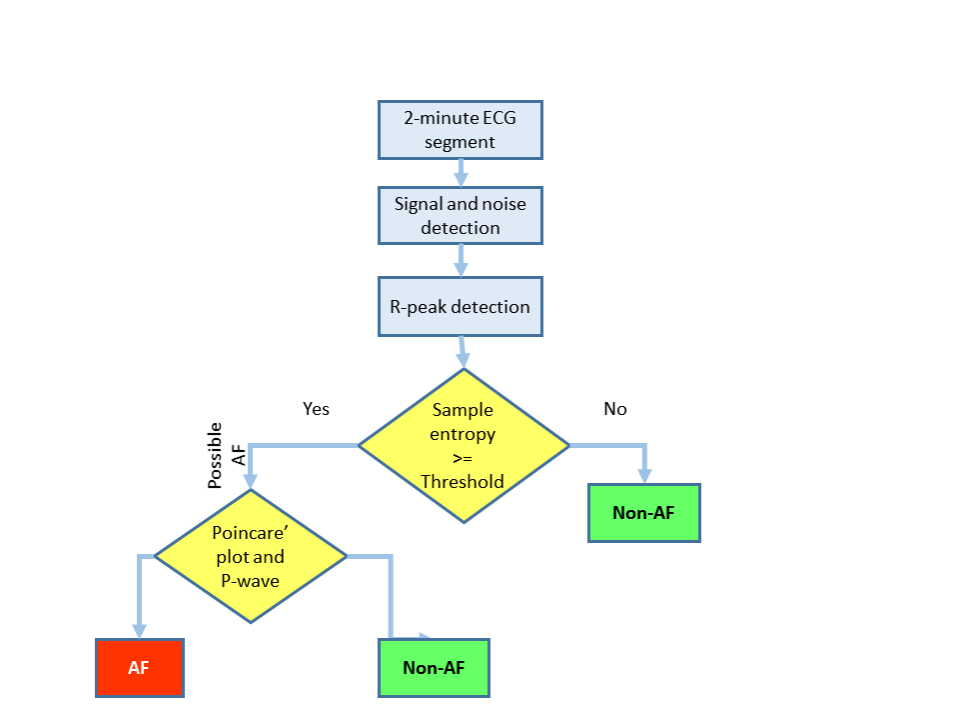
Flow diagram showing the steps used by the automated atrial fibrillation detection algorithm to classify AF status. AF: atrial fibrillation; ECG: electrocardiogram.

Continuous telemetry ECG recordings between 6 and 55 hours in length and with at least one readable ECG recording were divided into 2-minute segments, which were first analyzed for interpretable signal using automated signal and noise detection [13]. The 2-minute ECG segments without a predominance of noise were then analyzed with a novel R-wave detection method that detects QRS complexes using variable-frequency complex demodulation–based ECG reconstruction [14]. Next, the variability of R–R intervals was evaluated using sample entropy, a measure of randomness that is expected to be higher for patients with AF than those with normal sinus rhythm [15]. Based on the sample entropy calculated from the R–R intervals, an automated “initial screening” for AF was performed, where the “possible AF” status may include premature atrial and ventricular contraction segments as false-positive detections of AF. In order to differentiate increased R–R randomness from AF in contrast to R–R variability caused by premature atrial and ventricular beats, a novel premature beat detection step was added to the algorithm which only takes the “possible AF” segments determined by the sample entropy in the previous step [16]. Two approaches were used to differentiate premature atrial and ventricular beats from AF. First, Poincaré plots derived from the differences of heart rates were used to differentiate AF from premature atrial and ventricular beats as repeated triangular-shaped patterns were found for premature atrial and ventricular contractions in the Poincaré plot [9]. In addition to the Poincaré plots, P-waves were identified using a recently developed empirical mode decomposition–based algorithm [17]. Because AF is characterized by an absence of P-waves, but premature atrial and ventricular beats occur in the midst of sinus rhythms with P-waves that precede QRS complexes, high ratios of P-wave to R-wave were used to aid differentiation of premature beats from AF (low P-to-R ratio) [16]. Further, in order to increase the specificity of the AF detection algorithm, we a priori determined that the automated AF detection algorithm would identify a patient as having an AF episode only if 3 consecutive 2-minute ECG segments (6 minutes) were identified as containing continuous AF. The algorithm identified AF in one of the ECG leads, though an exploratory post-hoc analysis made all ECG leads available to the automated algorithm. A summary of the AF detection algorithm is shown in Figure 1.

### AF Algorithm Development and Validation

**Figure 2:**
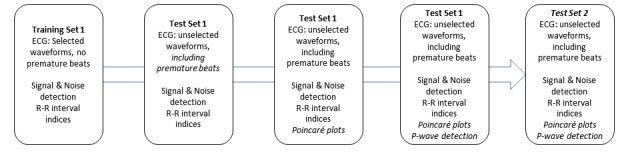
Flow diagram showing steps in the process of atrial fibrillation detection algorithm development, refinement, testing and validation. ECG: electrocardiogram.

AF detection algorithms were derived and validated in a stepwise manner (Figure 2). The AF detection algorithms using only automated noise detection and R–R sample entropy were first trained using selected waveforms without premature beats (training set 1, Round 1) and then validated (test set 1, Round 2) using randomly selected waveforms with and without AF. In order to determine the added value of premature beat detection, we added automated premature atrial and ventricular beat detection using Poincaré plots, and then added P-to-R-wave ratios to the algorithms tested in Rounds 1 and 2 and retested the algorithm in test set 1. In the final validation experiments (test set 2), we deployed the complete ensemble algorithm, which included noise detection, R–R sample entropy, and premature atrial and ventricular beat detection with Poincaré and P-wave detection, using 50 randomly chosen AF and non-AF waveforms. In total, 3 cohorts with 150 patients were evaluated using manual AF detection with results blinded to the deployment of the automated algorithm.

### Statistical Analyses

We evaluated agreement between the gold-standard review of telemetry ECG data by an expert ECG reader (DM) and other methods of AF detection including the automated AF detection algorithm, nurse charting of AF status, and ICD-9 codes using 2 × 2 contingency tables. Additionally, we performed a post-hoc exploratory analysis to evaluate the performance of previously described automated methods of AF detection in our test set—a statistical method [17] that used the root mean square of successive differences, Shannon entropy, and turning point ratio calculated from R–R intervals to automatically detect AF; and a method [18] that used the coefficient of sample entropy obtained from R–R intervals to determine the AF status. Sensitivity (true-positive rate), specificity (true-negative rate), positive (proportion of positive signals that are true positives) and negative predictive values (proportion of negative signals that are true negatives) were calculated for each AF algorithm with 95% confidence intervals using MedCalc (MedCalc Software). We calculated the average time between estimates of AF onset for the gold standard as compared with other methods and accuracy using SAS 9.4 (SAS Institute). Comparisons of accuracy were conducted with α=.05. All study procedures were deemed not human subjects research by the Boston University Medical Campus and University of Massachusetts Medical School Institutional Review Boards.

## Results

Among 58,976 ICU admissions within MIMIC III, we identified 14,831 admissions for adults with sepsis, among whom 2975 patients had ECG waveforms linked to clinical data. Three groups of 50 ECG waveforms from patients hospitalized with sepsis were randomly selected and evaluated iteratively through the automated AF detection algorithm. Characteristics of patients with waveforms selected for algorithm validation (test set 2) are shown in Table 1.

**Table 1:** Patient characteristics validation cohort (N=50).

Characteristic		Validation cohort
Age, mean (SD)		72.7 (12.9)
Sex, female, n (%)		19 (38)
**Race/ethnicity**		
	Black, n (%)	4 (8)
	White, n (%)	36 (72)
	Asian, n (%)	3 (6)
	Other, n (%)	1 (2)
	Unknown, n (%)	6 (12)
**Comorbidity score (Elixhauser–van Walraven), mean (SD)**		9.5 (6.7)
	Heart failure, n (%)	19 (38)
	Coronary artery disease, n (%)	18 (36)
	Valvular disease, n (%)	6 (12)
	Hypertension, n (%)	8 (16)
	Diabetes without complication, n (%)	6 (12)
	Diabetes with complication, n (%)	5 (10)
	Chronic pulmonary disease, n (%)	7 (14)
	Renal failure, n (%)	10 (20)
	Obesity, n (%)	2 (4)
**Source of infection**		
	Pulmonary infection, n (%)	23 (46)
	Genitourinary infection, n (%)	19 (38)
	Gastrointestinal infection, n (%)	4 (8)
	Skin/Soft tissue infection, n (%)	14 (28)
	Cardiovascular infection, n (%)	2 (4)
	Postprocedural infection, n (%)	14 (28)
	Unspecified septicemia, bacteremia, n (%)	22 (44)
	Other infection, n (%)	6 (12)
SOFA^a^ score, ICU^b^ day 1, mean (SD)		8.7 (4.0)

^a^SOFA: Sequential organ failure assessment.

^b^ICU: intensive care unit.

Results of the rounds of training and testing (validation) of the AF detection algorithm are shown in Tables 2-7. During the initial training round on waveforms selected for lack of noise, the accuracy of the R–R sample entropy algorithm for AF detection was 100% (Table 2). However, the algorithm performed more modestly when deployed in a separate set of unselected ECG waveforms (accuracy 76%; 95% CI 61%-87%; Table 3). Analysis of potential reasons for discrepancy between the automated algorithm and manual abstraction showed that the lower specificity and positive predictive value from the algorithm were due to false-positive AF detection in the setting of critically ill patients with a high burden of premature atrial beats. We next trained an algorithm to discriminate between premature beats and AF, first using Poincaré analysis (Table 4, 94% accuracy), and then with a combination Poincaré and P-wave detection (Table 5, 98% accuracy), both of which demonstrated improvement in accuracy (*P*=.02) as compared with models without the ability to detect premature beats. The high accuracy (94%, 95% CI 83%-99%) and low false-positive rate (96% specificity) were confirmed in a separate validation cohort (test cohort 2; Table 6). Exploratory analysis showed further improvement in algorithm performance with analysis of all available ECG leads for each patient (Table 7, 98% accuracy). The time of AF onset detected by the automated algorithm differed from the manual detection onset time by a median 30 minutes (25th-75th percentile 0-208 minutes), potentially related to the algorithm’s requirement for 3 consecutive 2-minute segments to be present to call AF.

**Table 2:** Results of training set 1^a^ for initial automated atrial fibrillation identification using signal and noise detection and R–R interval indices, from selected waveforms without premature beats.

Automated algorithm atrial fibrillation status	Manual atrial fibrillation status		
	Atrial fibrillation	No atrial fibrillation	Total
Atrial fibrillation	24	0	24
No atrial fibrillation	0	25	25
Total	24	25	49^b^

^a^The sensitivity, specificity, positive predictive value, negative predictive value, and accuracy were 100% (95% CI 86%-100%), 100% (95% CI 86%-100%), 100%, 100%, and 100% (95% CI 93%-100%), respectively.

^b^One patient had noise in the waveform tracings that were unable to be run through the algorithm due to lack of 3 consecutive 2-minute segments of majority noise-free time.

**Table 3:** Results of test set 1^a^ of automated atrial fibrillation detection using signal and noise detection and R–R interval indices only, from unselected waveforms.

Automated algorithm atrial fibrillation status	Manual atrial fibrillation status		
	Atrial fibrillation	No atrial fibrillation	Total
Atrial fibrillation	10	11	21
No atrial fibrillation	1	27	28
Total	11	38	49^b^

^a^The sensitivity, specificity, positive predictive value, negative predictive value, and accuracy were 91% (95% CI 59%-100%), 71% (95% CI 54%-85%), 48% (95% CI 35%-61%), 96% (95% CI 80%-99%), and 76% (95% CI 61%-87%), respectively.

^b^One patient had extensive noise in the waveform tracings that were unable to be run through the algorithm.

**Table 4:** Results of test set 1^a^ for automated atrial fibrillation using signal and noise detection and R–R interval indices, with added Poincaré plots to detect premature beats from unselected waveforms.

Automated algorithm atrial fibrillation status	Manual atrial fibrillation status		
	Atrial fibrillation	No atrial fibrillation	Total
Atrial fibrillation	11	4	14
No atrial fibrillation	0	34	35
Total	11	38	49

^a^The sensitivity, specificity, positive predictive value, negative predictive value, and accuracy were 100% (95% CI 72%-100%), 90% (95% CI 75%-97%), 73% (95% CI 52%-87%), 100%, and 92% (95% CI 80%-98%), respectively.

**Table 5:** Results of test set 1^a^ for automated atrial fibrillation using signal and noise detection and R–R interval indices, with added Poincaré, and P-wave indices to detect premature beats from unselected waveforms.

Automated algorithm atrial fibrillation status	Manual atrial fibrillation status		
	Atrial fibrillation	No atrial fibrillation	Total
Atrial fibrillation	11	2	12
No atrial fibrillation	0	36	37
Total	11	38	49

^a^The sensitivity, specificity, positive predictive value, negative predictive value, and accuracy were 100% (95% CI 72%-100%), 95% (95% CI 82%-99%), 85% (95% CI 59%-96%), 100%, and 96% (95% CI 86%-99%), respectively.

**Table 6:** Results of the test set 2^a^ for automated atrial fibrillation signal and noise detection and R–R interval indices, with added P-wave and premature beat detection, from unselected waveforms.

Automated algorithm atrial fibrillation status	Manual atrial fibrillation status		
	Atrial fibrillation	No atrial fibrillation	Total
Atrial fibrillation	23	1	24
No atrial fibrillation	2	24	26
Total	25	25	50

^a^The sensitivity, specificity, positive predictive value, negative predictive value, and accuracy were 92% (95% CI 74%-99%), 96% (95% CI 80%-100%), 96% (95% CI 77%-99%), 92% (95% CI 76%-98%), and 94% (95% CI 83%-99%), respectively.

**Table 7:** Exploratory analysis on test set 2^a^, evaluating effect of using all available leads (rather than only 1 lead shown in for automated atrial fibrillation detection.

Automated algorithm atrial fibrillation status	Manual atrial fibrillation status		
	Atrial fibrillation	No atrial fibrillation	Total
Atrial fibrillation	25	1	26
No atrial fibrillation	0	24	24
Total	25	25	50

^a^The sensitivity, specificity, positive predictive value, negative predictive value, and accuracy were 100% (95% CI 86%-100%), 96% (95% CI 80%-99.9%), 96% (95% CI 79%-99%), 100% (95% CI 76%-98%), and 98% (95% CI 89%-99.9%), respectively.

We also compared administrative codes (ICD-9; Table 8) and nurse cardiac rhythm annotation (Table 9) with manual AF identification. Compared with the gold-standard manual review of the ECG telemetry data, ICD-9 codes associated with AF showed 68% agreement (*P*=.002 vs automated algorithm) and nurse annotation of AF showed 80% agreement (*P*=.02 vs automated algorithm). Although timing of AF onset could not be estimated from ICD-9 codes, nurse charting of AF onset occurred a median of 56 minutes after gold-standard AF onset time (25th to 75th percentile of –13 to 705 minutes).

**Table 8:** Comparison of manual electrocardiographic detection of atrial fibrillation with ICD-9 codes for atrial fibrillation.^a^

ICD-9^b^ codes	Manual atrial fibrillation status		
	Atrial fibrillation	No atrial fibrillation	Total
Atrial fibrillation	16	7	23
No atrial fibrillation	9	18	27
Total	25	25	50

^a^The sensitivity, specificity, positive predictive value, negative predictive value, and accuracy were 64% (95% CI 43%-82%), 72% (95% CI 50%-88%), 70% (95% CI 53%-82%), 67% (95% CI 53%-78%), and 68% (95% CI 53%-80%), respectively.

^b^ICD-9: International Classification of Diseases, 9th edition.

**Table 9:** Comparison of manual electrocardiographic detection of atrial fibrillation with nurse electronic medical record heart rhythm annotation for atrial fibrillation.^a^

Nurse charting	Manual atrial fibrillation status		
	Atrial fibrillation	No atrial fibrillation	Total
Atrial fibrillation	24	9	33
No atrial fibrillation	1	16	17
Total	25	25	50

^a^The sensitivity, specificity, positive predictive value, negative predictive value, and accuracy were 96% (95% CI 80%-100%), 64% (95% CI 43%-82%), 73% (95% CI 61%-82%), 94% (95% CI 70%-99%), and 80% (95% CI 66%-90%), respectively.

Finally, we performed an exploratory analysis to evaluate the performance of 2 previously described AF detection algorithms [18,19] (Multimedia Appendices 1 and 2). Both previously described algorithms had numerically lower accuracy than our novel AF-detection ensemble (Dash et al [18]: 90% accuracy, 95% CI 78%-97%; Lake and Moorman [19]: 76% accuracy, 95% CI 62%-87%).

## Discussion

### Principal Findings

We developed, validated, and evaluated a novel, automated, accurate algorithm to detect AF from stored electronic health record ECG waveform data from telemetry recordings. We used a stepwise approach to algorithm development and demonstrated that the automated AF detection algorithm worked by first eliminating waveforms with noisy segments that impaired reliable rhythm assessment, next by discriminating premature atrial and ventricular beats that mimic the rhythm irregularity from AF, and finally by using R-wave variability algorithms to detect AF from 2-minute-long ECG segments. The automated algorithm demonstrated predictive values greater than 90% and detecting AF within a median 30 minutes of manual ascertainment. The automated algorithm showed favorable performance characteristics when compared with currently available standard methods of large-scale AF ascertainment, including diagnostic codes, nurse annotation of rhythm status recorded in the electronic medical record, and previously described automated AF detection approaches [18,19].

### Limitations

Our findings should be considered in light of study limitations. Data arose from a single center and diagnostic claims coding and nurse documentation of heart rhythm status may differ at other centers. Further testing of the performance of the automated AF detection algorithm in other settings and in comparison to other automated methods of AF detection, such as machine learning techniques, is certainly warranted. Strengths of this study include the manual validation of all key ECG segments by trained study personnel with oversight of an expert ECG reader, use of an algorithm that automates signal and noise detection, and the stepwise analysis quantifying improvement in algorithm performance when adding different features, which demonstrate the necessity of adding premature beat detection to an algorithm designed to detect AF in the setting of critical illness.

### Comparison With Prior Work

Few prior studies have evaluated automated algorithms for AF detection among critically ill patients. Moss et al [7] tested an algorithm using an ensemble of R–R interval time-series approaches previously developed from outpatient Holter rhythm monitoring [19] among 500 30-minute telemetry segments of ICU patients in a single center, and found sensitivity and positive predictive value of 89% and 99%, respectively. The method of AF detection by Moss et al [7] differed from our algorithm in multiple ways: we used automated noise detection to select evaluable ECG segments, required shorter ECG segments for analysis (2 minutes vs 10 minutes), and combined R–R time-series approaches (ie, sample entropy and Poincaré plot features) with P-wave characteristics in order to discriminate premature beats from AF. Accuracy of AF onset times were not reported in the Moss et al [7] ICU sample. Although we do not directly compare the algorithm described by Moss et al using MIMIC ECG data, use of an earlier iteration of the ensemble used by Lake and Moorman [19] showed less favorable accuracy within our cohort when compared with our novel algorithm. Results from our stepwise, iterative analysis of automated algorithm performance demonstrated the importance of incorporating strategies that could identify P-waves and differentiate premature atrial and ventricular beats from AF among critically ill patients with sepsis. Given differences in patient characteristics and validation strategies between Moss et al [7] and our study, further studies comparing different automated approaches to AF detection within an independent validation cohort are warranted.

In addition to determining accuracy of a novel, automated ECG detection algorithm for AF detection, we also evaluated existing methods of AF recognition within claims data ICD-9 codes and electronic medical record–based nurse annotation of heart rhythm. Compared with manual ECG review, ICD-9 codes were unable to identify AF timing and showed only modest performance (68% accuracy, 70% positive predictive value, and 67% negative predictive value) for correctly identifying cases of AF during the ICU stay. Nurse charting of heart rhythm status performed similar to ICD-9 codes for rhythm status determination, and although nurse charting allowed for timing of AF episodes [12], AF onset times from nurse-charted AF episodes differed from the gold-standard rhythm onset by approximately 1 hour. Thus, in our sample of patients with sepsis, automated AF detection was superior to current standard large-scale approaches to AF detection using electronic health record data. Prior studies validating ICD-9 codes for AF detection showed better performance than our sample [5], potentially because ECG data were available only from ICU in this study, rather than the entire hospitalization.

Multiple potential uses exist for an algorithm that can accurately read and identify AF from ECG waveform data from critically ill patients with sepsis. Our automated AF detection algorithm is a novel tool that facilitates the analysis of underutilized continuous waveform data currently housed in electronic data repositories, and allows AF to be studied using “big data” analytic approaches. Large-scale AF identification can be used in future studies to evaluate risk factors and triggers of AF, and to study long-term ramifications of subclinical AF occurring during acute illness such as sepsis. Because of the automated detection of ECG signal, noise, premature beats, and AF, the algorithms can also be adapted and scaled for rapid, real-time identification of AF among patients undergoing continuous ECG monitoring, including critically ill patients with complex ECG waveforms. The AF algorithm based on sample entropy is computationally more efficient than machine learning algorithms that require significant training data, and reports similar accuracy to machine learning methods not subjected to the additional challenge of high premature beat burdens met by the present algorithm among critically ill patients [20-22]. Furthermore, algorithm development was hypothesis driven, enabling us to understand the relative contributions of premature beats and ECG noise to overall AF detection performance. Despite the fact that the prevalence of AF in unselected ambulatory populations may be lower than in our sample of inpatients with sepsis, our AF detection approach with noise cancellation and premature beat discrimination may also be useful in ambulatory ECG data from Holter monitors [23] and ECG data from wearable devices, as these devices are also frequently affected by motion and noise artifact.

### Conclusions

We derived and validated an automated algorithm that detects an ECG signal, eliminates segments corrupted by noise artifact, and can discriminate AF from other causes of irregular R–R intervals such as premature atrial and ventricular beats. The automated algorithm performed with higher accuracy than currently available methods for large-scale AF detection, including ICD codes and nurse charting of heart rhythm status from data in the electronic health record. Further studies can use the algorithm to identify AF in large-scale electronic health record data to facilitate studies of risk factors and triggers of AF, as well as long-term complications of subclinical AF during acute illness.

## Appendix

Multimedia Appendix 1 Results of an alternate automated atrial fibrillation detection method (Dash et al [18]; Test set 2).

Multimedia Appendix 2 Comparison of automated atrial fibrillation detection with COSEn-based method (Lake and Moorman [19]; Test set 2).

## Abbreviations

AF: atrial fibrillation
ECG: electrocardiogram
ICD-9: International Classification of Diseases, 9th edition
MIMIC: Medical Information Mart for Intensive Care
