# Supplementary material for: Development and Validation of an Automated Algorithm to Detect Atrial Fibrillation Within Stored Intensive Care Unit Continuous Electrocardiographic Data: Observational Study
Source: JMIR Cardio. 2021 Feb 15;5(1):e18840. doi: 10.2196/18840 (PMC8411425; doi:10.2196/18840)
Supplement: Multimedia Appendix 1 [file cardio_v5i1e18840_app1.docx]

| ***Automated Algorithm***  ***(Statistical Method)***  ***AF Status*** | ***Manual AF Status*** | | |
| --- | --- | --- | --- |
|  | ***Atrial fibrillation*** | ***No Atrial fibrillation*** | ***Total*** |
| **Atrial fibrillation** | 25 | 5 | 30 |
| **No Atrial fibrillation** | 0 | 20 | 20 |
| **Total** | 25 | 25 | 50 |

S. Dash, K. Chon, S. Lu, and E. Raeder, “Automatic real time detection of atrial fibrillation,” Annals of biomedical engineering. 2009: 37(9):1701–1709.

| **Sensitivity** | **100% (95% CI, 86-100%)** |
| --- | --- |
| **Specificity** | **80% (95% CI, 59-93%)** |
| **Positive Predictive Value** | **83% (70-92)** |
| **Negative Predictive Value** | **100 %** |
| **Accuracy** | **90% (95% CI, 78-97%)** |
