# Supplementary material for: Development and Validation of an Automated Algorithm to Detect Atrial Fibrillation Within Stored Intensive Care Unit Continuous Electrocardiographic Data: Observational Study
Source: JMIR Cardio. 2021 Feb 15;5(1):e18840. doi: 10.2196/18840 (PMC8411425; doi:10.2196/18840)
Supplement: Multimedia Appendix 2 [file cardio_v5i1e18840_app2.docx]

| ***Automated Algorithm***  ***(COSEn-based)***  ***AF Status*** | ***Manual AF Status*** | | |
| --- | --- | --- | --- |
|  | ***Atrial fibrillation*** | ***No Atrial fibrillation*** | ***Total*** |
| **Atrial fibrillation** | 25 | 12 | 37 |
| **No Atrial fibrillation** | 0 | 13 | 13 |
| **Total** | 25 | 25 | 50 |

D. E. Lake and J. R. Moorman, “Accurate estimation of entropy in very short physiological time series: the problem of atrial fibrillation detection in implanted ventricular devices,” American Journal of Physiology-Heart and Circulatory Physiology. 2011: 300(1): H319–H325.

| **Sensitivity** | **100% (95% CI, 86-100%)** |
| --- | --- |
| **Specificity** | **52% (95% CI, 31-72%)** |
| **Positive Predictive Value** | **68%** |
| **Negative Predictive Value** | **100%** |
| **Accuracy** | **76% (95% CI, 62-87%)** |
